# The Role of Immunomodulatory Receptors in the Pathogenesis of HIV Infection: A Therapeutic Opportunity for HIV Cure?

**DOI:** 10.3389/fimmu.2020.01223

**Published:** 2020-07-02

**Authors:** Hui Chen, Maha Moussa, Marta Catalfamo

**Affiliations:** ^1^Department of Microbiology and Immunology, Georgetown University School of Medicine, Washington, DC, United States; ^2^CMRS/Laboratory of Immunoregulation, National Institutes of Allergy and Infectious Diseases, Bethesda, MD, United States

**Keywords:** CD8 T cells, HIV, checkpoint receptors, checkpoint inhibition therapy, HIV pathogenesis

## Abstract

Immune activation is the hallmark of HIV infection and plays a role in the pathogenesis of the disease. In the context of suppressed HIV RNA replication by combination antiretroviral therapy (cART), there remains immune activation which is associated to the HIV reservoirs. Persistent virus contributes to a sustained inflammatory environment promoting accumulation of “activated/exhausted” T cells with diminished effector function. These T cells show increased expression of immunomodulatory receptors including Programmed cell death protein (PD1), Cytotoxic T Lymphocyte Associated Protein 4 (CTLA4), Lymphocyte activation gene 3 (LAG3), T cell immunoglobulin and ITIM domain (TIGIT), T cell immunoglobulin and mucin domain containing 3 (TIM3) among others. More importantly, recent reports had demonstrated that, HIV infected T cells express checkpoint receptors, contributing to their survival and promoting maintenance of the viral reservoir. Therapeutic strategies are focused on viral reservoir elimination and/or those to achieve sustained cART-free virologic remission. In this review, we will discuss the immunological basis and the latest advances of the use of checkpoint inhibitors to treat HIV infection.

## Introduction

The immune system is designed to regulate the balance between immunity and tissue damage. This process is mediated by receptor-ligand interactions that provide positive or negative co-stimulatory signals. Particularly, the inhibitory receptors function as “brakes” for the adaptive immune response and serve as checkpoints to control tissue immunopathology. The signaling through these inhibitory receptors regulates the immune response and, in case of failure of pathogen elimination these pathways have profound impact on the host homeostasis contributing to the disease. Because of their impact during immune responses, checkpoint receptors became major therapeutic targets in a wide spectrum of diseases including cancer and chronic viral infections such as HIV (1–3).

Different to other human chronic viral infections, HIV targets the immune system altering immune mechanisms, and is characterized by chronic immune activation, the main player in the pathogenesis of the disease (4–6). In the context of suppressed HIV replication by combination antiretroviral therapy (cART), immune activation is associated with poor clinical outcomes such as the non-AIDS defining illnesses which are today the leading cause of mortality and morbidity in patients with HIV infection.

Persistent infection contributes to a sustained inflammatory environment promoting accumulation of activated T cells. HIV-specific T cells and other virus specific T cells such as EBV, CMV, and influenza show an immune activated phenotype (4, 6–8). The main feature of these activated T cells is an “exhausted phenotype” suggested by increased expression of one or co-expression of several immunomodulatory receptors including PD1, CTLA4, LAG3, TIGIT, TIM3, CD160 among others. Exhausted HIV-specific T cells have diminished effector function and fail to control viral replication (9–25).

The essential role that HIV/SIV-specific cytotoxic T lymphocytes (CTL) play to control viral replication was demonstrated by *in vivo* depletion of CD8 T cells that resulted in lack of viral control during acute and chronic Simian Immunodeficiency Virus (SIV) infection (26–30). In addition, in human infection, viral escape mechanisms emerge early during infection and are contributing factors for the failure of CD8 T cell mediated immunity (8, 31, 32).

HIV-specific CD4 T cells are important in the immunity against HIV, however their role is hampered by being the major targets of HIV/SIV infection (13, 33–38). In addition, CD4 T cells are the main cell type harboring the HIV/SIV reservoirs in tissues and recent evidence determined that HIV latently infected CD4 T cells express checkpoint receptors promoting viral persistence (22, 23, 39).

This evidence suggests that immune therapeutic approaches directed to block immune checkpoint receptors will have two-level effect on the viral reservoir and HIV-specific T cell responses. In this review, we will discuss the latest advances in this area.

## The Role of Checkpoint Receptors in HIV Infection

**Figure 1:**
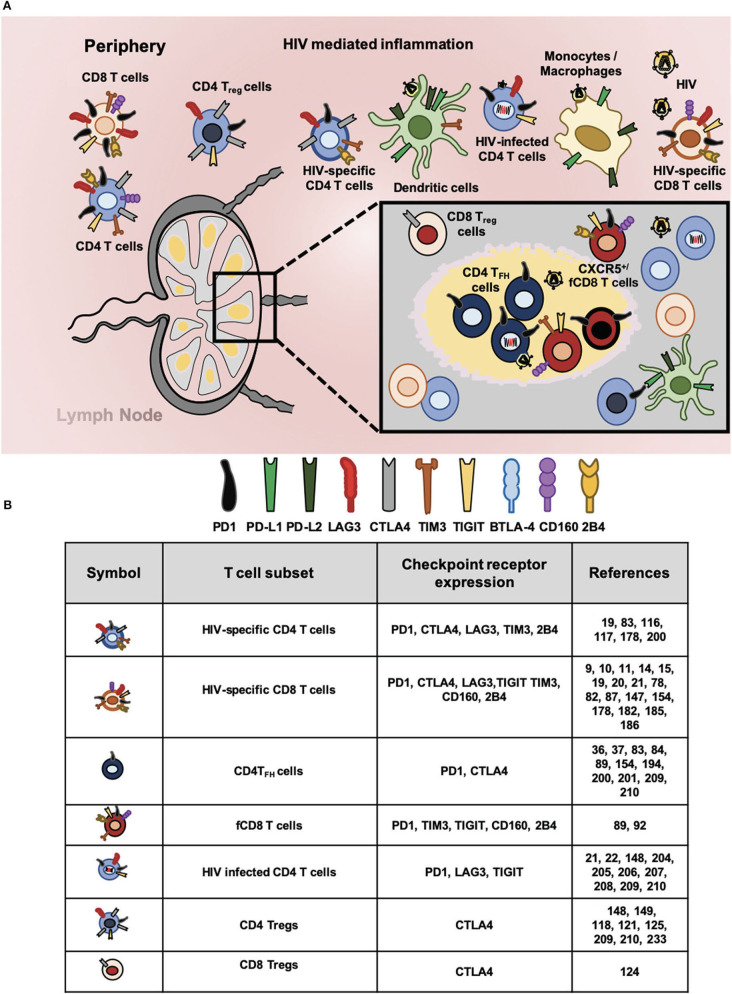
Checkpoint receptors expression in HIV-specific T cells and latently infected CD4 T cells. **(A)** Chronic immune activation and inflammation are the hallmark of HIV infection. In this context, cells of innate and adaptive immune system became dysfunctional and express aberrant levels of checkpoint receptors that hampers HIV-specific responses. Proportionally to antigen abundance and persistence, several checkpoints receptors became upregulated particularly in different T cell subsets. In circulation and lymphoid tissues, total CD4 and CD8 T cells; regulatory CD4 T (Treg) and CD8 (Treg) T cells; follicular helper CD4 T (T_FH_), and follicular CD8 T (fCD8 T) cells; HIV-specific CD4 and CD8 T cells. In addition, HIV infected CD4 T cells express surface checkpoint receptors such as Programmed cell death protein 1 (PD1), Cytotoxic T lymphocyte antigen 4 (CTLA4), Lymphocyte activation gene 3 protein (LAG3), T cell immunoglobulin and mucin domain receptor 3 (TIM3), T cell immunoreceptor with immunoglobulin and ITIM domains (TIGIT), B and T lymphocyte attenuator (BTLA), CD160, and 2B4. Antigen presenting cells (APC, mainly monocytes/macrophages and dendritic cells) upregulate checkpoints receptors that bind to the ligands expressed by lymphocytes. Accordingly, Programmed cell death protein ligand 1 (PD-L1) and ligand 2 (PD-L2) along with other inhibitory receptors are upregulated by APCs regulating T cell mediated immunity against HIV. **(B)** Expression of checkpoint receptors by T cell subsets. The wide spectrum of T cell subsets that express checkpoint receptors suggest their blockade will promote latency reversal and elimination by invigorated HIV-specific T cells.

The checkpoint receptors PD1 and CTLA4 are the most extensively studied *in vitro* and *in vivo* in the context of HIV/ SIV infection. The checkpoint receptors such as LAG3, TIGIT, TIM3, and others are also expressed by T cells and their role in the pathogenesis of the infection is not well-defined. More importantly, the observation that several checkpoint receptors are co-expressed by latently infected CD4 T cells, suggest new roles of these molecules in viral persistence and their potential to be used as reversal agents have emerged in the last few years (Figure 1).

### PD1 (CD279)

PD1 was discovered by Ishida et al. in 1992 and its function in regulating the immune response was elucidated few years later when the *Pdcd1* deficient mice was developed and showed a lupus-like autoimmune disease (40–42). PD1 binds to two ligands, PD-L1 (B7-H1) and PD-L2 (B7-DC). PD-L1 is expressed by a variety of hematopoietic cells including, T and B cells, DCs, macrophages, and non-hematopoietic cells including mesenchymal stem cells, lung epithelial cells, vascular endothelium, liver non-parenchymal cells, placental synctiotrophoblasts, and keratinocytes (1, 43, 44). In contrast, PD-L2 expression is more restricted to antigen presenting cells such as dendritic cells, macrophages, and germinal center B cells and its expression is modulated by inflammatory signals (45–47). The most characterized function of the PD1/PD-L1 pathway is tuning T cell responses, however the wide range of cells that express PD-L1 suggests other unexplored functions in regulating immune responses. The effects of PD-L2 interaction with PD1 is not well-defined. PD-L2 binds with higher affinity to PD1 indicating that may compete with PD-L1 (48). In addition, PD-L2 expression is inducible by Th2 cytokines and may play a role in regulating Th2 responses (48, 49). However, PD-L2 has shown inhibitory effects on proliferation and cytokine secretion including IFNγ suggesting a role in modulating also Th1 responses (50–52).

PD1 signaling occurs through its intracytoplasmic domain that contains an immunoreceptor tyrosine-based inhibitory motif (ITIM) and an immunoreceptor tyrosine-based switch motif (ITSM) (53, 54). PD1 exerts its inhibitory function when signals in combination with TCR engagement. The tyrosine residue located in the ITSM is phosphorylated recruiting protein tyrosine phosphatases. The tyrosine phosphatase SHP2 interferes with molecules involved in TCR signaling (ZAP70, PKCθ, and CD3ζ) and downstream pathways of the TCR-CD28 signaling including phosphoinositide 3-kinase (PI3K)–AKT, and RAS. In addition, it was shown that in absence of SHP-2, SHP-1 phosphatase can be recruited and promote similar effects on T cells (55, 56). The interference of these pathways leads to inhibition of T cell activation, effector function, proliferation, and survival (45, 53, 57–64).

PD1 is expressed by many subsets of T cells including memory CD4 and CD8 T cells, T regulatory cells (Tregs), T follicular helper (T_FH_), and T follicular regulatory (T_FR_) cells (65–67). In addition, expression of PD1 has been also shown in B cells, NK cells and myeloid cells suggesting that blockade of this receptor has wide impact on the immune response (47). In addition to attenuating T cell activation in many subsets of T cells, new roles for PD1 have been reported. Particularly, it has been shown that its expression by CD4 T_FH_ cells is required for their positioning inside the B cell zone and for the secretion of IL-21. In contrast, PD1 signaling prevents activated T cells from entering into the follicles (68).

PD1 has been mainly studied in CD8 T cells in the context of chronic infection and cancer. In an acute infection, PD1 expression is modulated by TCR signaling and its expression is reduced after elimination of the pathogen (47, 69). In contrast, in chronic infections and cancer, continuous antigen stimulation leads to sustained expression of PD1 and diminished effector function (70–74).

The effector CD8 T cells that develop in these conditions undergo a distinct “exhausted” transcriptional and epigenetic program than CD8 effector T cells developed during an acute infection. Recent studies had shown that “exhausted” CD8 T cells are in fact a heterogenous population, and some of these CD8 T cells possess stem-like properties defined by expression of the transcription factor T-cell factor 1 (TCF1) (75, 76). These CD8 T cells are responsible to maintain the immune response during chronic infection, and in contrast to exhausted cells they have self-renewal properties and expand during PD1 therapy (75). The features of “exhausted” T cells, in terms of their transcriptional, epigenetic, metabolic, and others properties, are discussed in depth by 18 experts in the field in a recent point-of-view article that highlights their heterogeneity and pathways driving their differentiation (77).

#### PD1 Expression in HIV Infection

In human chronic infections such as HIV infection, PD1/PD-L1 pathway is one of the checkpoint receptors that has been extensively studied in an attempt to restore T cell mediated immunity against HIV. PD1 expression in CD8 T cells is associated with the levels of viremia and disease progression, and its expression is only partially reduced after successfully suppressed viremia by cART treatment (18, 78–81). Exhausted HIV-specific CD8 T cells have diminished effector function, proliferative capacity, and increased susceptibility to undergo apoptosis. This evidence suggests that this pathway plays a critical role in the failure of CD8 T cells to mediate viral clearance (9, 10, 78).

The antigen dependency in the expression of PD1 by CD8 T cells during chronic HIV infection was further demonstrated in a study of a longitudinal cohort of HIV infected patients. HIV-specific CD8 T cells for those epitopes that underwent mutational escape during infection showed decreased expression of PD1. In contrast, HIV-specific CD8 T cells with TCR specificities for conserved epitopes expressed higher levels of PD1 and showed poor effector function (82). These data confirmed that chronic TCR stimulation plays a major role in the sustained expression of PD1 and the development of exhausted CD8 T cells in HIV infection.

Other important aspect of checkpoint receptors in the context of HIV infection is that they are expressed by several subsets of infected CD4 T cells. For instance, CD4 T_FH_ cells are the major HIV cell reservoir and are localized in the B cell follicles. This micro-compartmentalization of the viral reservoir inside the lymphoid organs has drawn the focus toward CD8 T cell immunity against HIV mediated by CD8 follicular (fCD8) T cells. fCD8 T cells are defined by expression of CXCR5, and they have ability to migrate into the B cell follicle where the ligand of CXCR5 is expressed. In addition, another important subset is the tissue resident memory CD8 T (CD8 T_RM_) cells which are also present in lymphoid organs (83–86).

In SIV/HIV infection, most of the virus-specific CD8 T cells are localized in extrafollicular area around the B cell follicles (87, 88). In contrast, CXCR5^+^ fCD8 T cells are enriched in the follicles and their frequency is associated with local immune activation rather than viral replication (89). fCD8 T cells expressed high levels of PD1 and some co-expressed TIGIT (89). fCD8 T cells, showed diminished polyfunctionality measured by cytokine secretion that was restored by blockade of the PD1/PD-L1 pathway. Studies in SIV infection, showed that fCD8 T cells localized inside and around the follicles and have cytotoxic potential (90, 91).

The role of PD1 in fCD8 T cells is not well-understood. The recent evidence that PD1 signaling is involved in preventing activated T cells to traffic into the B cell follicles suggests that PD1 blockade can be a strategy to promote trafficking of HIV-specific T cells to the sites of viral reservoirs. In this regard, *in vivo* blockade of PD1 led to expansion of SIV-specific fCD8 T cells co-expressing cytolytic molecules in the lymph nodes, although the study did not evaluate the frequency of these cells inside the follicles (24).

The potential of fCD8 T cells to traffic into the B cell follicles where the HIV infected CD4 T_FH_ cells are localized suggests that therapies enhancing differentiation, and effector function of fCD8 T cells can be an important intervention to be considered in cure strategies (92, 93).

Another important subset of CD8 T cells involved in tissue immune surveillance is the tissue resident memory T cells. CD8 T_RM_ cells were first described in peripheral tissues and represent the first line of defense during memory immune responses. These T cells do not circulate and their transcriptional profile varies between tissue resident cells from different tissues suggesting tissue-specific properties of these cells (94). Recent reports have identified populations of CD4 T_RM_ cells and CD8 T_RM_ cells in lymph nodes, they express CD69, a tissue residency marker, and inhibitory receptors including PD1 indicating a physiological role of PD1 in balancing tissue damage during immune responses (95–97).

In the context of HIV infection, higher frequencies of HIV-specific CD8 T_RM_ are present in the lymphoid nodes from HIV infected elite controllers compared with HIV infected progressors highlighting a central role for these cells in limiting viral replication (85, 86, 98, 99). In addition, CD8 T_RM_ cells from the lymph nodes expressed higher levels of CD69 and their transcriptional profile was consistent with tissue residency rather than activation. While most of the CD69^+^ CD8 T_RM_ cells are localized in the extrafollicular areas, a smaller fraction of these cells are found inside the B cell follicles suggesting that they can migrate into the sites of HIV viral reservoir (85, 86). This evidence has led to the hypothesis that enhancing trafficking into the follicle of CD8 T cells and/or promoting differentiation into fCD8 T cell subset can be an efficacious strategy for viral control/elimination.

### CTLA4 (CD152)

CTLA4 belongs to the CD28 family of proteins and its expression is upregulated on T cells upon TCR stimulation. In contrast to conventional T cells, Tregs constitutively express CTLA4 suggesting a prominent role of this checkpoint receptor in Treg function.

CTLA4 competes with CD28 for the binding to CD80 and CD86 expressed on the surface of antigen presenting cells attenuating T cell activation (100, 101). In Tregs, CTLA4 is stored in a vesicular intracellular compartment and upon TCR activation is released at the immunological synapse (102). The regulated secretion of CTLA4 requires calcium influx and is modulated by adaptor molecules including T cell receptor-interacting molecule (TRIM), Linker for activation of X cells (LAX) and others (103–106). CTLA4 YVKM motif interacts with tyrosine phosphatase SHP-2 and serine-threonine phosphatase protein phosphatase 2A inhibiting TCR signaling (60, 105, 107, 108). The signaling pathway of CTLA4 is largely inconclusive, and has been suggested that CTLA4 cytoplasmic domain function is not to transmit downstream regulatory signals but rather control the turnover, cellular localization, and to regulate its secretion at the immunological synapse and control the binding of CD28 to CD80 and CD86 shared ligands (109–111).

#### CTLA4 Expression in HIV Infection

The role of CTLA4 in the context of HIV infection has been mostly described in CD4 T cells, and its expression is increased in both total and HIV-specific CD4 T cells. The role of CTLA4 in the pathogenesis of infection is suggested by the positive association with viral loads, and depletion of CD4 T cells (13, 112–116). In addition, CD4 T cells from HIV infected progressors were more likely to co-express multiple checkpoint receptors (PD1, CTLA4, and TIGIT), and at higher levels to that observed in elite controllers (117, 118). In contrast to CD4 T cells, HIV-specific CD8 T cells show lower expression of CTLA4 suggesting a more prominent role of this checkpoint receptor in CD4 T cells (13, 112–115, 117).

In Tregs, CTLA4 plays a major role in their regulatory function. In the context of SIV/HIV infection, Tregs have two-side function, while they can exert beneficial effects on reducing immune activation, they limit viral clearance (119–122). Accordantly, Tregs are present at the sites of viral replication such as lymph nodes, spleen, and gut associated lymphoid tissues, and their frequency is correlated with tissue SIV RNA levels (123). In addition, an accumulation of CD8 Tregs (CD25^+^FoxP3^+^CTLA4^high^) with suppressive effector function has been observed in lymphoid and colorectal mucosal tissues from viremic SIV infected rhesus macaques (RM) (124). Similar observations were made in humans in which Tregs expressed higher levels of CTLA4 compared with Tregs from individuals with suppressed viremia by cART (125). This evidence suggests that manipulating suppressor function by targeting CTLA4 and/or depletion of Tregs could be considered as a new approach for cure strategies (119).

### LAG3 (CD223)

Lymphocyte activation gene 3 (LAG3, CD223) belongs to the immunoglobulin superfamily and it is expressed by conventional T lymphocytes, Tregs, NK cells and plasmacytoid DCs upon activation (126, 127). The extracellular region of LAG3 protein consists of four immunoglobulin-like domains (D1–D4) with high homology and higher binding affinity to MHC than the CD4 co-receptor (128). In T cells, LAG3 expression is upregulated upon TCR activation and exerts its immunomodulatory function mainly via MHC dependent TCR inhibition (128–131). LAG3 binds to CD3/TCR complex, suppressing calcium flux, cytokine secretion, and proliferation (132). In contrast, the blockade of the D2 domain (outside of MHC binding site) of LAG3 molecule improved T cell function suggesting possible involvement of other ligands for LAG3 particularly in case of CD8 T cells and NK cells (133). Several alternative ligands have been proposed for LAG3 including, Galectin-3, liver sinusoidal endothelial cell lectin (LSECtin) and fibrinogen-related protein (FGL-1) a soluble factor (134–136).

The intracytoplasmic region of LAG3 contains three domains: the first is a serine-phosphorylation site, the second region is a KIEELE motif, and the third a glutamic acid-proline (EP) repeats (137). In effector CD4 T cells, the KIEELE motif is essential for its inhibitory function, however, the downstream signaling molecules involved are not well-defined, and if this domain is participating in the signaling in other cells such as CD8 T cells is largely unknown (137). In addition, tissues metalloproteases can cleave LAG3 between the D4 transmembrane domain and the transmembrane domain, generating a soluble LAG3 and this mechanism can regulate T cell responses (138).

#### LAG3 Expression in HIV Infection

In the context of SIV/HIV infection, the expression of LAG3 has been reported to be elevated in T cells and iNKT cells in the tissues and blood, and its expression is associated with levels of viremia and disease progression (139, 140). In the context of suppressed viremia, T cell expression of LAG3 alone or in combination with PD1 has been associated with cardiovascular disease. While the mechanism of this association is largely unknown, it may reflect the state of chronic immune activation which is associated to increased risk of cardiovascular disease (19, 139–146). Similar expression of LAG3 has been described in HIV- and CMV-specific T cells from patients with HIV infection suggesting distinct roles of LAG3 in regulating HIV-specific immune responses (147, 148). In addition, expression of LAG3 has been also described in a subset of Tregs that co-express CD49b. These regulatory T cells are called Type I Tregs, and they secrete large amounts of IL-10. In patients with HIV infection, increase frequencies of Type I Tregs were associated with disease progression (149).

Co-expression of LAG3 and other inhibitory receptors including PD1 and TIGIT are enriched in latently infected CD4 T cells from HIV infected patients with suppressed viral loads suggesting that immune checkpoint receptors contribute to viral persistence. While the role of LAG3 in the pathogenesis of HIV is not well-understood, this evidence suggests that blockade of LAG3 can have an impact in cell mediated immunity and the viral reservoir (22, 148, 150).

### TIGIT

TIGIT is a member of the immunoglobulin superfamily and belongs to the family of poliovirus receptors. TIGIT, CD226, and CD96 are expressed by NK cells and some T cells subsets including effector T cells, memory T cells, and T_FH_ (127, 151–153). TIGIT inhibitory function is mediated by binding to its ligand the poliovirus receptor (PVR, CD155), and poliovirus receptor-relate 2/Nectin-2 (PVRL2, CD112) which are present on the surface of APCs and non-hematopoietic cells including tumor cells. TIGIT binds to the same ligands than CD226 (DNAM-1) and CD96 (Tactile), and together with CD226 promotes a positive co-stimulatory signal while CD96 and TIGIT transduce inhibitory signals. In addition, TIGIT inhibit the costimulatory signals of CD226 either by competition, binds with higher affinity to CD155; or by binding in cis to CD226, and blocks the costimulatory signals (127, 151, 154).

The intracellular domain of TIGIT contains an ITIM and an immunoglobulin tail tyrosine (ITT)-like motif. In mice, phosphorylation of the tyrosine residue in either motif is sufficient for transduce the inhibitory signal. In contrast in humans, phosphorylation of the tyrosine residue in ITIM or the ITT-like motif seems to be essential, and the contribution of either motif in the inhibitory signal remain undefined. TIGIT binding to CD155 promotes recruitment of SH2-containing tyrosine phosphatase SHP1 inhibiting phosphoinositide 3-kinase (PI3K) and mitogen-activated protein kinase (MAPK). Of note is that most of the signaling pathways have been described in NK cells, and in these cells, TIGIT inhibit both cytotoxicity and cytokine secretion (152, 155, 156). In T cells, TIGIT mediates its regulatory function by downregulation of the TCR complex and inhibition of PLCγ. While these effects of TIGIT leads to inhibition of T cell activation, proliferation, and effector function, also, promotes survival of these inhibited T cells by upregulation of γc cytokine receptors (IL-2, IL-7, and IL-15) and Bcl-xL (157).

#### TIGIT Expression in HIV Infection

In the context of HIV/SIV infection, a large portion of virus-specific CD8 T cells express TIGIT and have diminished effector function (21, 154). Increased frequencies of CD8 T cells expressing TIGIT alone or with PD1 are associated with disease progression. Moreover, *in vitro* blockade of TIGIT and the PD1/PD-L1 pathway restored HIV-specific CD8 T cell function suggesting that blockade of TIGIT in combination with other checkpoint receptors that may be a potential therapy to enhance T cell immunity against HIV (21).

Expression of TIGIT and PD1 by CD4 T cells have been associated with immune activation and increased risk of cardiovascular diseases in HIV infected patients as measured by 2-year change in coronary artery calcium. However, the mechanism in which TIGIT can contributes to cardiovascular disease is largely unknown (158). TIGIT is expressed in other T cell subsets from HIV infected patients. For instance, γδ T cells expressed TIGIT and its expression is associated with immune activation and pro-inflammatory function (159). In NK cells from HIV infected individuals, expression of TIGIT is correlated with disease progression and NK cell dysfunction (160). Although the role of TIGIT is not well-defined in HIV infection, TIGIT ligands such as PVR are increased by CD4 T_FH_ cells in lymph nodes from HIV infected patients compared to heathy donors suggesting it may play a role in viral persistence (161).

### TIM3 (CD366)

TIM3, also known as Hepatitis A virus cellular receptor 2 (HAVCR2), belongs to T cell immunoglobulin and mucin domain (TIM) family. TIM3 is expressed in Th1 and Th17 CD4 T cells, CD8 T cells, and it is constitutively expressed by Tregs (162–165). In addition, TIM3 expression has been observed in NK cells and myeloid cells including monocytes, dendritic cells and macrophages (166, 167). TIM3 has several ligands including galectin-9, phosphatidylserine (PtdSer), high-mobility group box 1 (HMGB1), and carcinoembryonic antigen related cell adhesion molecule 1 (Ceacam-1) (168–171). Therefore, the cell type and the ligand that TIM3 engages determines the inhibitory mechanism. TIM3 binding to galectin has inhibitory function by inducing cell death of autoreactive Th1 T cells in an experimental autoimmune encephalomyelitis (EAE) model (169). Binding of TIM3 to PtdSer leads to the uptake of apoptotic cells and this pathway promotes cross-presentation of antigens by dendritic cells (172). HMGB1 which normally binds to DNA released from dying cells induces activation of innate cells and secretion of inflammatory cytokines, and TIM3 binding interferes with this pathway (171). Ceacam-1 binds to TIM3 in *cis* and *trans* and both type of interactions drive inhibitory signals of TIM3.

TIM3 lack of classical inhibitory motifs, and its cytoplasmic domain has five conserved tyrosine residues that can be phosphorylated by Src kinases or interleukin inducible T cell kinase. In absence of interaction with its ligand, TIM3 is bound to the HLA-B associated transcript 3 (Bat3) and recruits the catalytic form of Lck, in this state TIM3 does not interferes with TCR signaling. In contrast, when TIM3 binds to the ligands such as galectin 3 and Ceacam-1, triggers phosphorylation of the tyrosine Y256 and Y263 and this results in the release of Bat3 allowing the recruitment of SH2 domain-containing proteins and inhibiting TCR signaling (127, 168, 173–175).

In addition, TIM3 interacts with the receptor phosphatases CD45 and CD148 disrupting the immunological synapse, suggesting additional mechanisms involved in the inhibitory function of this checkpoint receptor. In human CD8 T cells, TIM3 is localized in lipid rafts and its blockade allows formation of a stable immunological synapse (176).

#### TIM3 Expression in HIV Infection

In the context of HIV infection, polymorphism in *HAVCR2*, the gene that encodes TIM3, was partially associated with the susceptibility to HIV infection (177). In HIV/SIV acute infection, T cells expressed higher levels of TIM3 that is reduced following initiation of cART (178–180). In addition, expression of Bat3, the regulator of TIM3 activity, is reduced suggesting an active inhibitory signal by TIM3 during HIV infection (173). Accordantly, TIM3^+^ HIV-specific CD8 T cells are dysfunctional and have defective degranulation (80, 178, 179, 181–184). Blockade of TIM3 restore proliferation and functional capacity of HIV-specific CD8 T cells (178, 184, 185).

In CD4 T cells, TIM3 mainly regulates Th1 responses (162, 165, 169). In acute and chronically HIV infected patients, TIM3 expression in CD4 T cells was associated with immune activation markers such as CD38, viral loads, and CD4 T cell depletion (80, 178). CD4 T cells express higher levels of TIM3 than CD8 T cells and its expression was significantly reduced after viral suppression by cART (179). In HIV-specific CD4 T cells, TIM3 upregulation is associated with diminished effector function including cytokine production, proliferation, and cell survival (178, 180, 181, 186). This evidence suggests that TIM3 can regulate CD4 and CD8 T cell responses in the context of infection.

### Other Checkpoint Receptors

Checkpoint receptors are expressed by distinct T cell subsets and their mode of action determines the wide arrays of effects in T cell immune response and their therapeutic potential. Other checkpoint receptors that have been shown to have inhibitory effects on HIV-specific T cell responses are CD160 and 2B4 (15, 20, 147). CD160 is expressed by HIV-specific CD8 T cells and its blockade improved proliferation and cytokine production (15). In addition, combination of checkpoint inhibitors such as anti-PD1 and anti-2B4 showed a synergistic effect in enhancing the proliferation capacity of HIV-specific CD8 T cells (147). In HIV elite controllers, a subset of HIV-specific CD8 T cells that co-express 2B4 and CD160 has cytolytic capacity, as measured by perforin expression suggesting that these receptors may have distinct role in regulating T cell responses in the context of HIV infection (20).

Therefore, the levels and the variety of checkpoint receptors expressed by HIV-specific T cells reflects immune activation and determines the extend of T cell exhaustion differentiation in the context of HIV infection (15, 116, 147).

## The Role of the Checkpoint Receptors in Viral Persistence and Elimination

The main obstacle to cure HIV infection is elimination of the viral reservoirs which are established very early during infection (187). The viral reservoirs consist of replication-competent and defective form of viruses that accumulate and persist at several anatomical sites, preferentially the lymphoid tissues (188–192). Several reports have shown that despite successfully viral suppression by cART, there is a high heterogeneity among individuals in terms of the frequency of latently infected cells and residual viremia. Accordantly, viral blips are suggestive of higher HIV reservoirs, highlighting potential challenges in the role of these factors in cure strategies (193–196).

The immune system, particularly HIV-specific CD8 T cells are confronted with several challenges to eliminate the viral reservoirs. Latently infected cells contain viruses with escape mutations selected at early stages of infection (197). The presence of defective proviruses contributes to the immune activation, and creates a diversion in the elimination of replication competent viruses by CD8 T cells (189, 195). Chronic immune activation and exhaustion of HIV-specific T cells leads to immune dysfunction and failure of viral elimination (6, 198, 199).

The compartmentalization of the reservoirs inside the B cell follicles in the lymph nodes is a contributing factor for viral persistence. In the follicles, follicular dendritic cells can harbor infectious virus for extended periods of time promoting infection of newly recruited CD4 T_FH_ cells. In addition, to gain access to the follicles, acquisition of CXCR5 expression by HIV-specific CD8 T cells is required representing another factor that contributes to HIV persistence (83, 84, 87, 88, 200–203).

Recent evidence had demonstrated that multiple immune checkpoint receptors are preferentially expressed by latently infected cells presenting an additional challenge to overcome by the virus-specific CD8 T cells (21, 22, 148, 204–207). In patients with suppressed viremia by cART, CD4 T cells co-expressing several checkpoint receptors such as PD1, TIGIT, and LAG3 contain ten-times more HIV-DNA than the checkpoint receptor negative CD4 T cell counterparts (21, 22, 208). Moreover, CTLA4 expression was observed in a subset of CD4 T cells that share a Treg phenotype and harbor replication competent viruses suggesting that many T cell subsets and checkpoint receptor dependent mechanisms are involved in viral persistence (209, 210).

Approaches targeting the viral reservoirs are under intensive investigation and blockade of checkpoint receptors can be one of them to “unshield” HIV infected cells and enhance effector function of HIV-specific CD8 T cells at tissue sites (23, 211).

## The Impact of Targeting Immune Checkpoint Receptors in HIV-specific Responses and the Viral Reservoir

**Figure 2:**
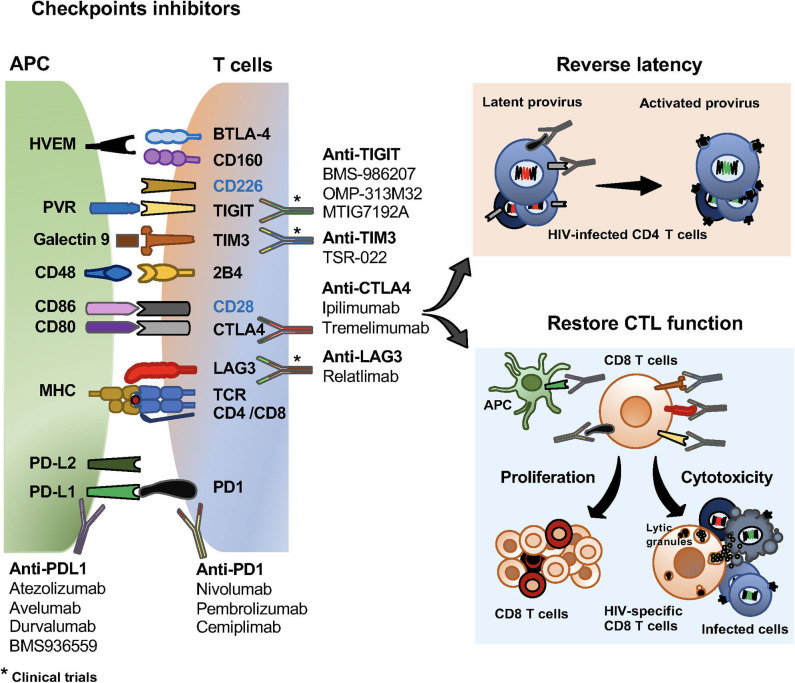
Checkpoint inhibitors and their effects on HIV specific T cells and the viral reservoir. Checkpoint inhibitors in clinical trials for the treatment of malignancies and HIV infection. Monoclonal antibodies blocking PD1, PD-L1, CTLA4, LAG3, TIGIT and TIM3 currently use in clinical trials. Effects of checkpoint receptors in reversing latency of the viral reservoir, and restoring CD8 T cell proliferation and cytotoxic functions.

The compelling evidence about the role of the checkpoint receptors in HIV-specific T cell responses and viral persistence has driven pre- and clinical studies to assess the effects of immune checkpoint blockade for the treatment of HIV infection (summarized in Table 1, Figure 2).

**Table 1:** Effects of immune checkpoint receptors blockade in SIV/HIV specific responses.

**Target molecule**	**Viral reservoirs**	**SIV/HIV specific immunity (CD4, CD8 and B cells) and other immune responses**
PD1	***In vitro:*** *Pembrolizumab* reduced latency in CD4 T cells (206, 207). *Pembrolizumab* and bryostatin, enhanced HIV production in CD4 T cells from HIV infected patients (207). *Nivolumab* had no consistent impact on viral reactivation of CD4 T cells from HIV infected patients (212). ***In vivo (SIV):*** Anti-PD1 (clone EH12-2132/2133) treatment reduced the viral reservoir (24). Anti-PD1 (clone EH12-1540) not reported (16, 213, 214). Anti-PD1 (Clone 1B8) not reported (215). Anti-PD1 (Human/rhesus chimeric antibody) alone or with vesatolimod had no statistical impact on the viral reserviors (216). Anti-PD1 (Human/rhesus chimeric antibody) and anti-CTLA4 dual blockade reduced the total and intact SIV DNA in CD4+ T cells as well as SIV DNA and RNA level in B cell follicles in lymph nodes (217). ***In vivo*** **(HIV):** *Pembrolizumab* administered HIV infected patient (case study) induced latency reversal (206). *Pembrolizumab* (case study) treatment reduced total and integrated HIV DNA and cell associated HIV RNA (207). *Pembrolizumab* (phase I trial) not reported (218). *Nivolumab* (case study) transient increased in cell associated HIV-DNA (219). *Nivolumab* (case study) decrease in cell associated HIV DNA (220). *Nivolumab or Pembrolizumab* no consistent changes in cell associated HIV RNA or DNA in CD4 T cells (221)	***In vitro:*** *Pembrolizumab* (MK-3475) enhanced cytokine production upon antigen stimulation (184) *Nivolumab* not reported (212). ***In vivo*** **(SIV):** Anti-PD1 (clone EH12-2132/2133) enhanced SIV-specific CD8 T cell function and reduced type I IFN signaling (24). Anti-PD1 (clone EH12-1540) enhanced anti-HIV specific CD8 T cell and B cell responses, reduced type I IFN signaling (16, 213, 214). Anti-PD1 (clone EH12-1540) reduced plasma viral load (213). Anti-PD1 (Clone 1B8) transiently elevated viral load (215). Anti-PD1 (Human/rhesus chimeric antibody) treatment did not improve viral suppression after ART cessasion (216). Anti-PD1 (Human/rhesus chimeric antibody) and anti-CTLA4 dual blockade did not affect virus specific CD8 response and plasma viral load (217). ***In vivo*** **(HIV):** *Pembrolizumab* not reported (206). *Pembrolizumab* (phase I trial) not reported (218). *Nivolumab* (case study): slightly increased IFNγ secreting CD8 T cells (219). *Nivolumab* (case study) not reported (220). *Nivolumab or Pembrolizumab* small HIVGag-specific CD4 and CD8 T cell responses. No changes in EBV/CMV or TCR stimulated T cell responses. No changes in HIV-specific antibodies (221)
PD-L1	***In vitro:*** Anti-PD-L1 (Clone 12A4) not reported (21) Anti-PD-L1 (Clone MIH1) not reported (116). *BMS-936559* No consistent impact on viral reactivation of CD4 T cells from HIV infected patients (212). ***In vivo*** **(SIV):** *Avelumab* not reported (222, 223) *BMS-936559* not reported (225) ***In vivo (HIV):*** *BMS-936559* (NCT02028403) did not affect viral reservoirs (224).	***In vitro:*** Anti-PD-L1 (Clone 12A4) enhanced viral specific CD8 T cell response (21). Anti-PD-L1 (Clone MIH1) promoted HIV- specific CD4 T cell proliferation (116). *BMS-936559* not reported (212). ***In vivo (SIV):*** *Avelumab* together with rhIL-15 expanded a subset of polyfunctional CD8 T cells and potential to traffic (223). *Avelumab* slightly delayed viral rebound after ART interruption (222). *BMS-936559* induced delayed viral rebound in 4 of 8 RMs (225) ***In vivo (HIV):*** *BMS-936559* (NCT02028403) increased cytokine production in some HIV infected patients without malignancy (224).
CTLA4	***In vitro:*** Anti-CTLA4 (BNI3) not reported (13, 226). ***In vivo*** **(SIV):** Anti-CTLA4 (MDX-010) not reported (227, 228). ***In vivo*** **(HIV):** *Ipilimumab* (case report) increased cell-associated RNA in one HIV-infected patient (229).	***In vivo*** Anti-CTLA4 (BNI3) restored HIV-specific CD4 T cell function (13). Anti-CTLA4 (BNI3) blocked Treg mediated inhibition of actin polymerization, DC maturation, viral infection (226). ***In vivo*** **(SIV):** Anti-CTLA4 (MDX-010) decreased HIV RNA in lymph node and enhanced HIV-specific effector T cell function (227). Anti-CTLA4 (MDX-010) induced T cell activation and viral replication especially at mucosal site (228). ***In vivo (HIV):*** *Ipilimumab* (NCT03407105) showed inconsistent change on HIV RNA in viremic HIV- infected patients without malignancy (230).
TIM3	***In vitro:*** Soluble TIM3 glycoprotein and anti-TIM3 (Clone 2E2) not reported (178). Anti-TIM3 (Clone 344,823) not reported (184).	***In vitro:*** Soluble TIM3 glycoprotein and anti-TIM3 (Clone 2E2) promote virus specific T cell proliferation (178). Anti-TIM3 (Clone 344,823) enhanced cytokine production and proliferation of virus specific CD8 T cells, especially in combination with anti-PD1 (184).
TIGIT	***In vitro:*** Anti-TIGIT (Clone 11G11 and 23G8) not reported.	***In vitro:*** Anti-TIGIT (Clone 11G11 and 23G8) increased viral specific CD8 T cell response when using with anti-PD-L1 (Clone 12A4) (21).

*Unless mentioned specifically, case reports and clinical trials are HIV- infected patients with malignancy*.

### Pre-clinical Studies

#### *In vitro* Studies

Recent studies using an *in vitro* infection model of mDCs-CD4 T cells co-culture, had shown the contribution of PD1 engagement in the establishment of HIV latency. In these culture conditions, blockade of PD1/PD-L1 pathway with pembrolizumab (anti-PD1) led to a modest but significant decrease in latently infected cell numbers (206). Pembrolizumab showed a synergistic effect when used in combination with a latency reversing agent bryostatin without enhancing T cell activation (207). In contrast, other report had shown small effects on viral replication in an *ex-vivo* stimulation of CD8 depleted PBMCs with CD3/CD28 in the presence of mAb BMS-936559 (anti-PD-L1) or Nivolumab (anti-PD1) (212). New studies are evaluating the effects of blockade of multiple checkpoint receptors. A recent report, showed that reversal of HIV latency was achieved by blockade of several checkpoint receptors PD-1, CTLA4, TIM3, and TIGIT without T cell stimulation, and this effect was significantly higher than using latent reversal agents such as vorinostat and bryostatin (231).

Therapeutic target of Tregs has been also considered in HIV cure strategies because they are susceptible to HIV infection and has regulatory functions on other cells such as HIV-specific T cells (119). In SIV/HIV infection viral DNA was detected in Tregs (209, 232–234). In co-culture experiments of Tregs and conventional CD4 T cells, Tregs reduced DC mediated viral infection of CD4 T cells. This effect was mediated by Treg inhibition of DC maturation, and actin polymerization that prevented the delivery of infectious viruses at the DC-T cell synapse. These effects were reversed by CTLA4 blockade (226). Moreover, *in vitro* CTLA4 blockade promoted HIV-specific CD4 T cell proliferation and cytokine production (13, 235). While Tregs can have both detrimental and beneficial effects in the context of HIV infection, new studies are ongoing to assess cure strategies targeting Tregs (119).

#### Checkpoint Inhibitor Treatment in SIV Infected RM

##### PD1/PD-L1 pathway

The blockade of the PD1/PD-L1 pathway is one of the most studied in the context of SIV infection as a single treatment or in combination with cytokines and other checkpoint inhibitors.

In chronically SIV infected macaques, administration of anti-PD1 (mouse anti-human clone EH12-1540) was well-tolerated, and enhanced anti-SIV immunity and viral control by improving SIV-specific CD8 T cell and B cell function (16, 213–215). Anti-PD1 treatment in combination with cART, reduced immune activation and improved memory B cell function (16, 214). In addition, PD1 blockade using a non-human primate anti-PD1, clone EH12-2132/2133, enhanced the expansion of CXCR5^+^ CD8 T cells expressing perforin and granzyme B, and reduced regulatory T cells resulting in decreased viral replication. These data suggested that blockade of PD1 have a potential effect in enhancing trafficking of CD8 T cells into the B cell follicles (24).

We and others have shown that administration of anti-PD-L1 antibodies (Avelumab and BMS-936559) in RM infected with SIV was safe and well-tolerated (222, 225). The effects on viral replication after discontinuation of cART showed that anti-PD-L1 (BMS-936559) led to a delay in the viral rebound although the effect was transient (225). In the Avelumab study, three animals were treated and showed no significant effect on viral rebound (222).

These pre-clinical studies blocking the PD1/PD-L1 pathway as single therapy suggested that combination therapies may achieve better outcomes in controlling viral replication. In this regard, rhIL-15 is a candidate to use with a checkpoint inhibitor. In *in vitro*, and *in vivo* studies in non-human primates, IL-15 promoted homeostatic proliferation, survival, and enhancing effector function of NK and memory CD8 T cells (236–241).

In SIV infected RM, while transient proliferation of CD8 T cells was observed, IL-15 did not enhanced CD4 T cell reconstitution at the lymph nodes and mucosal sites (242). A recent study used rhIL-15 in combination with anti-PD-L1 (Avelumab) in SIV infected RM. The treatment was safe and well-tolerated, and promoted expansion of CXCR3^+^PD1^−/low^ SIV_Gag_-specific CD8 T cells with effector function capacity and potential to traffic in peripheral tissues. However, no effect on viral rebound was observed after cART discontinuation (223). These data suggest that regimens promoting sustained expansion of this population may be more efficacious. In addition, the CXCR3^+^PD1^−/low^ SIV_Gag_-specific CD8 T cells may be targeted as a potential source of more effective virus-specific CD8 T cells, and this combination can be used as a tool to enhanced functionality of virus-specific cell preparation for adoptive T cell transfer therapies.

##### CTLA4 blockade

Accordantly to the *in vitro* data reported, *in vivo* CTLA4 blockade mostly impacts conventional CD4 T cells and Tregs. The effects of *in vivo* blockade of CTLA4 are difficult to interpret because the constitutive expression of this receptor by CD4^+^CD25^high^FoxP3^+^ Tregs, and conventional CD4^+^CD25^−^ T cells express CTLA4 upon activation. Studies aiming to block Treg mediated immune suppression through CTLA4 were carried out using an anti-CTLA4 mAb (human antibody, MDX-010) in virally suppressed SIV infected RM (227). CTLA4 blockade facilitated viral control in lymphoid tissues when compared with cART only treated macaques (227). In contrast, another study showed that CTLA4 blockade during primary and chronic SIV infection, increased T cell immune activation and viral replication in tissues particularly at the mucosal sites suggesting that blockade of CTLA4 can have wider effects on T cells responses (228).

Administration of anti-CTLA4 in combination with PD1 blockade in SIV infected macaques, drove expansion of effector T cells, increased of plasma viremia and decreased cell associated DNA in B cell follicles indicating that this treatment facilitated viral activation and promote T cell function (217, 244).

CTLA4 and other checkpoint has been assessed in the context of vaccines. CTLA4 blockade in cynomolgus macaques administered at the time of an HIV envelope vaccine enhanced antibody responses. This study opens new avenues for the transient use of checkpoint receptors blockade as adjuvants in the context of HIV vaccines (245).

### Clinical Studies

There are few reported and on-going clinical studies using checkpoint receptors to treat HIV infection (Table 2, Figure 2). Most of the published studies in HIV infected patients receiving checkpoint inhibitors are those for treatment of malignancies, and in addition to safety and efficacy, the effects on the viral load and HIV-specific responses are being evaluated.

**Table 2:** Clinical trials of immune checkpoint blockade in HIV infected patients.

**Trial ID**	**Number of patients**	**Agent/Target molecule**	**Patient population**	**Phase**	**Clinical parameter measured**
**TRIALS FOR TREATMENT OF HIV INFECTION**					
NCT03407105 (230)	24	Ipilimumab CTLA-4	Viremic HIV patient (viral load 1,000–100,000 copies/ml, CD4 count ≥100 cells/ul)	I	Tolerability, CD4 count, viral load, drug pharmacokinetics
NCT03367754	60	Pembrolizumab PD-1	Suppressed HIV infected patients (T cell count 100–350 cells/mm^3^)	I	Tolerability, CD4 CD8 count, viral load, CTL function, PD-1 expression
NCT03239899	20	Pembrolizumab PD-1	Suppressed HIV infected patients (plasma HIV RNA <40 copies/ml, CD4 count above 350 cells/μl)	I	HIV-specific T cell responses, CSF HIV-specific responses and PD-1 expression
NCT02028403 (224)	8	BMS-936559 PD-L1	HIV patients with suppressed viremia (CD4 ≥ 350 cells/μl, plasma HIV-1 RNA <40 copies/ml)	I	CD4 count, viral load, *ex vivo* proliferation, and CTL functions
NCT03787095	45	Cemiplimab PD-1	HIV patients with suppressed viremia (CD4 ≥ 350 cells/mm^3^)	I/II	*Ex vivo* HIV-specific CTL function
NCT04223804	50	ABBV-181 (Budigalimab) PD-1	HIV patients with suppressed viremia (CD4 ≥ 500 cells/mm^3^)	I	Safety/tolerability, pharmacokinetics, and pharmacodynamics
**TRIALS FOR TREATMENT OF MALIGNANCIES IN PATIENTS WITH HIV INFECTION**					
NCT02408861	96	Ipilimumab + Nivolumab CTLA-4+PD-1	Suppressed HIV infected patients with HIV associated relapsed or refractory classical Hodgkin lymphoma or solid tumors (metastatic or cannot be removed by surgery)	I	Tolerability, tumor responses, viral load, CD4 count
NCT03304093	30	Nivolumab PD-1	Suppressed HIV infected patients with advanced NSCLC (viral load <200 copies/ml)	II	Diseases control rate, tumor survival, and responses rate, tolerability, viral load
NCT02595866 (218)	30	Pembrolizumab PD-1	Virally suppressed HIV infected with advance cancer including AIDs defining Hodgkin lymphoma, Kaposi sarcoma, and non-AIDs defining anal cancer, advance skin squamous cell carcinoma et al. (CD4 ≥ 100 cells/μl, viral load <200 copies/ml)	I	Tumor responses, CD4 count, viral load
NCT03094286 (243)	20	Durvalumab PD-L1	HIV infected patients with solid tumor (CD4 > 350 cells/mm3l)	II	Feasibility of treatment, response rate, survival

The safety profile of anti-PD1 (Pembrolizumab) was assessed in an open-label, non-randomized, phase 1 study in HIV infected patients (*n* = 30) with advanced cancer. Pembrolizumab treatment was shown to be safe in this patient population (CD4 counts > 100 cells/μl and viral loads <200 copies/ml) (218). Similar observations were noted in a systematic review of published literature about anti-PD1 and anti-CTLA4 antibodies used for the treatment of advanced-stage cancer in patients with HIV infection (*n* = 73). This study showed similar safety characteristics to that observed in the non-HIV infected individuals (246).

In the studies in which virological and immunological parameters were monitored, the reported effects were not consistent among them. A case report study showed that administration of Nivolumab (anti-PD1) in one HIV infected patient with advanced non-small cell lung cancer (NSCLC) led to a mild and transient increase in cell-associated HIV DNA (219). Another case report using Nivolumab for treatment of lung cancer, reported transient increase in plasma viremia, accompanied by enhanced viral-specific CD8 T cell function. After multiple doses, a decrease in HIV cell-associated DNA was observed (220). In contrast, other studies reported none or inconsistent changes in cell associated RNA/DNA when blocking the PD1/PD-L1 pathway (221, 224). In addition, in patients with lung cancer treated with three or four doses of Nivolumab, no changes in plasma viremia and CD4 T cell counts (247). These inconsistencies are likely due to the small number of patients and differences in the methods to assess the viral reservoir.

The observation that multiple doses of anti-CTLA4 (Ipilimumab) for treatment of metastatic melanoma in an HIV infected patient, led to increase in cell-associated unspliced HIV RNA and a decreased in levels of viremia suggested that Ipilimumab may play a role in the elimination of latently infected CD4 T cells (229). Similarly, another individual treated with a single dose of Nivolumab showed increase of cell-associated HIV RNA in CD4 T cells without changes in HIV DNA or viremia (206). These observations indicate that PD1 blockade in combination with other checkpoint receptors can have an effect on reactivation of the viral reservoir.

Only two phase I studies have been published using checkpoint inhibitors to treat HIV infection and some more are ongoing (Table 2). A phase I (NCT02028403) randomized, double-blind, placebo-controlled, dose-escalating study using BMS-936559 was reported (224). In this study, a single dose administration of anti PD-L1 mAb (BMS-936559, 0.3 mg/kg) in 6 HIV-infected participants on cART and HIV RNA between 0.4 and 40 copies/mL by single copy assay (SCA) showed that treatment was well-tolerated and there was an increased trend of the proportion of HIV_Gag_-specific CD8 T cells in two individuals. No changes were noted in the SCA over the 28 days post-infusion. This study was discontinued due to retinal toxicity observed in a parallel non-human primate study (224).

The second study is a dose escalating clinical trial using Ipilimumab, a fully human anti-CTLA4 antibody in HIV infected patients with uncontrolled viremia (NCT03407105). Ipilimumab monotherapy appeared to be safe and well-tolerated with mild to moderate adverse events at all doses tested. Treatment resulted in no significant changes in CD4 counts. The impact on HIV RNA varied among the patients, trending to a decrease in the lower dose group and an increased trend in the higher dose groups was observed (230).

The immunological and virological outcome of the treatment with checkpoint receptors in HIV infected patients remains unclear and more comprehensive studies are needed. The evidence that several checkpoint receptors may play a role in the pathogenesis of HIV infection suggests that combination therapy may achieve better outcomes (19, 23, 140, 211, 248). In this regard, ongoing clinical studies are evaluating the safety and efficacy of anti-LAG3, anti-TIGIT and anti-TIM3 antibodies alone or in combination with anti-PD1 for treatment of various types of malignancies (2). These checkpoint receptors are enriched in latently infected cells and the safety data of these studies will determine the potential use of these checkpoint receptors in HIV infection as reversal agents and/or to enhance HIV-specific T cell responses.

## Immune Checkpoint Inhibitors Treatment for Malignancies in HIV Infected Patients

HIV infected patients are at higher risk of developing both AIDS defining malignancies as well as non-AIDS defining malignancies (249–253). Particularly, non-AIDS malignancies are the leading cause of morbidity and mortality (249–251, 254–257). Patients with HIV infection and malignancies are at higher risk of cancer related mortality than the general population (256, 258). The mechanisms by which HIV increased risk of development of cancer and its impact in the disease progression are largely unknown. Studies directed to understand the effect of HIV in the tumor microenvironment and its impact on treatment response to checkpoint inhibitors are lacking.

Patients with HIV infection were generally excluded from the clinical trials with immune checkpoint blockade, because of the safety concerns and the potential adverse outcomes (259–262). Case reports and clinical trials focusing on various types of malignancies for HIV infected patients started to emerge since 2017 (218, 263–273).

A recent systemic review summarized all published cases until April 2018, including 73 HIV-infected patients that received several immune checkpoint inhibitor treatments (including Pembrolizumab, Nivolumab, and Ipilimumab) for advanced cancer, evaluating the safety, antitumor efficacy as well as the impact on HIV viral load and CD4 counts. The review showed the immune checkpoint inhibitors are generally safe and well-tolerated for HIV infected patients with advanced cancers, with a similar adverse events profile to that observed in uninfected cancer patients. Nine percentage of patients reported grade 3 or higher immune-related toxicities, mostly occurring in patients receiving Ipilimumab as part of their regimen. The response rate to tumor is also comparable between HIV-infected and non-HIV-infected cancer patients. Ninety-three percentage of the patients starting with undetectable viral load remained suppressed during immune checkpoint inhibitor treatment. Among patients with a detectable HIV viral load, five out of six patients had a decrease in viral load (246, 274). Other report, showed stable viral loads and CD4 cell counts in 176 HIV infected patients reported in the literature (275).

The data reported from a phase I clinical trial (NCT02595866) evaluating the safety and tolerability of anti-PD1 antibody Pembrolizumab in 30 HIV patients with various advanced cancer including AIDS defining malignancy such as non-Hodgkin lymphoma, Kaposi sarcoma, and non-AIDS defining malignancy including anal cancer, advanced skin squamous cell carcinoma and others. Pembrolizumab was safe and showed similar tumor response rate to the cancer patients without HIV infection. In this study, an unexpected lethal herpesvirus-associated multicentric Castleman disease with polyclonal B-cell lymphoproliferation was reported for one patient with Kaposi sarcoma indicating a risk of using Pembrolizumab in this patient population. In addition, Pembrolizumab administration did not induce changes in CD4 counts and HIV viral loads in 77% of the patients. Seven patients showed viral blips although lower than 400 copies/ml (218). So far current data support the extended use of immune checkpoint inhibitors in HIV infected patients. More clinical trials focusing on HIV infected population are ongoing and data will be reported in the next years to better understand the efficacy and anti-tumor response of immune checkpoint blockade for the treatment of cancer in patients with HIV infection (Table 2).

## Conclusions and Future Directions

The use of checkpoint inhibitors for the treatment of HIV infection is currently under intensive investigation. Blockade of checkpoint receptors restore immune function, and new opportunities had surfaced from the data suggesting that checkpoint inhibitors act as latency reversal agents to “unshield” HIV in infected cells.

The use of a checkpoint blockade in chronic HIV/SIV infection had shown transient effects in T cell mediated immunity compared to the success observed in the treatment of malignancies.

While T cell immunity in the setting of cancer and chronic HIV infection share similar immune mechanisms, the exhaustion in HIV-specific T cell seems to be difficult to reverse compared to the exhausted cells in the setting of cancer. Studies had shown that transcriptional and epigenetic programs are imprinted in exhausted cells, and this differentiation program limit their response to checkpoint receptor blockade. Therefore, duration of infection, chronic immune activation, and expression of several checkpoint receptors suggest that HIV-specific T cells may have limited plasticity. How to overcome these challenges requires further investigation, whether T cells at early stages of infection will be more susceptible to reverse exhaustion, or combination of several checkpoint receptors will achieve better results. Future studies directed to address transcriptional and epigenetic regulation of the differentiation pathways will unravel new targets to complement checkpoint blockade therapies that may be more advantageous in the setting of chronic infections.

## Author Contributions

HC, MM, and MC wrote the review and edited the text. HC prepared the tables. MM prepared the figures. MC composed the manuscript. All authors listed have made intellectual contribution and approved it for submission.

## Conflict of Interest

The authors declare that the research was conducted in the absence of any commercial or financial relationships that could be construed as a potential conflict of interest.
